# Supplementary material for: Focus group discussions on low-flow oxygen and bubble CPAP treatments among mothers of young children in Malawi: a CPAP IMPACT substudy
Source: BMJ Open. 2020 May 12;10(5):e034545. doi: 10.1136/bmjopen-2019-034545 (PMC7228516; doi:10.1136/bmjopen-2019-034545)
Supplement: Supplementary data [file bmjopen-2019-034545supp001.pdf]

Appendix 1: Example facilitator guide for focus group discussions with mothers whose child was on oxygen

Thank you all for joining us for this discussion. You have been invited to participate in this discussion because your child participated in a study where they were randomized to receive either bubble CPAP or oxygen therapy for severe pneumonia. We wanted to better understand your thoughts on treatment for pneumonia in young children and your experiences with oxygen.

1. Had you seen or heard about oxygen therapy before your child was hospitalized at Salima District Hospital?
    a. If so, please discuss what you had seen or heard.
    b. Had you heard about bubble CPAP, the other treatment used in the study?
2. What is your understanding of what oxygen therapy is?
    a. Where did you learn about oxygen therapy?
        i. PROBE: Can you identify who at the hospital provided you with information or was able to answer your questions?
    b. Do you feel like you received enough information or teaching about oxygen therapy?
        i. When did this teaching occur?
        ii. PROBE: Did you learn about oxygen therapy before or after your child was started on treatment?
3. How did you feel about your child receiving oxygen therapy at the beginning?
    a. What concerns did you have?
        i. PROBE: Did you hope your child would be in the oxygen therapy or bubble CPAP arm of the study? Why?

   b. Did your feelings about oxygen therapy change during the hospitalization?
      i. PROBE: Can you describe what it felt like to see your child on oxygen therapy?
4. What are the benefits of oxygen therapy for your child?
   a. PROBE: Can someone give me an example of a way that oxygen therapy may benefit a child with pneumonia?
5. Do you have any concerns that oxygen therapy can harm your child?
   a. If so, please explain.
   b. Do you think others in the community would have concerns about oxygen therapy?
6. What are other types of treatments for pneumonia?
   a. Are they better or worse? Why?

Thank you for your participation in this discussion today. This information will help us design future educational materials to help providers talk to mothers and caregivers about bubble CPAP and treatment for severe pneumonia. Please let us know if you have any questions. *[Clarify any misconceptions if any came up during the discussion.]*
