# Supplementary material for: Focus group discussions on low-flow oxygen and bubble CPAP treatments among mothers of young children in Malawi: a CPAP IMPACT substudy
Source: BMJ Open. 2020 May 12;10(5):e034545. doi: 10.1136/bmjopen-2019-034545 (PMC7228516; doi:10.1136/bmjopen-2019-034545)
Supplement: Author's manuscript [file bmjopen-2019-034545.draft_revisions.pdf]

BMJ Open is committed to open peer review. As part of this commitment we make the peer review history of every article we publish publicly available.

When an article is published we post the peer reviewers' comments and the authors' responses online. We also post the versions of the paper that were used during peer review. These are the versions that the peer review comments apply to.

The versions of the paper that follow are the versions that were submitted during the peer review process. They are not the versions of record or the final published versions. They should not be cited or distributed as the published version of this manuscript.

BMJ Open is an open access journal and the full, final, typeset and author-corrected version of record of the manuscript is available on our site with no access controls, subscription charges or pay-per-view fees (http://bmjopen.bmj.com).

If you have any questions on BMJ Open's open peer review process please email info.bmjopen@bmj.com

BMJ Open

Acceptability of low-flow oxygen and bubble CPAP treatments among CPAP IMPACT caregivers in Malawi

Journal:	*BMJ Open*
Manuscript ID	bmjopen-2019-034545
Article Type:	Original research
Date Submitted by the Author:	30-Sep-2019

Complete List of Authors:	Sessions, Kristen; McGaw Medical Center of Northwestern University, Pediatrics<br>Ruegsegger, Laura; University of North Carolina System, Project Malawi<br>Mvalo, Tisungane; University of North Carolina Project Malawi, ; University of North Carolina at Chapel Hill, Department of Pediatrics<br>Kondowe, Davie; University of North Carolina Project Malawi<br>Tsidya, Mercy; University of North Carolina Project Malawi<br>Hosseinipour, Mina C.; University of North Carolina Project Malawi<br>Lufesi, Norman; Ministry of Health, Acute Respiratory Infection Unit<br>Eckerle, Michelle; Cincinnati Children's Hospital Medical Center, Pediatrics<br>Smith, Andrew; University of Utah<br>McCollum, Eric ; Johns Hopkins School of Medicine

Keywords:	focus groups, developing countries, respiratory tract diseases, Child, continuous positive airway pressure, hypoxia

SCHOLARONE™ Manuscripts

I, the Submitting Author has the right to grant and does grant on behalf of all authors of the Work (as defined in the below author licence), an exclusive licence and/or a non-exclusive licence for contributions from authors who are: i) UK Crown employees; ii) where BMJ has agreed a CC-BY licence shall apply, and/or iii) in accordance with the terms applicable for US Federal Government officers or employees acting as part of their official duties; on a worldwide, perpetual, irrevocable, royalty-free basis to BMJ Publishing Group Ltd ("BMJ") its licensees and where the relevant Journal is co-owned by BMJ to the co-owners of the Journal, to publish the Work in this journal and any other BMJ products and to exploit all rights, as set out in our licence.

The Submitting Author accepts and understands that any supply made under these terms is made by BMJ to the Submitting Author unless you are acting as an employee on behalf of your employer or a postgraduate student of an affiliated institution which is paying any applicable article publishing charge ("APC") for Open Access articles. Where the Submitting Author wishes to make the Work available on an Open Access basis (and intends to pay the relevant APC), the terms of reuse of such Open Access shall be governed by a Creative Commons licence – details of these licences and which Creative Commons licence will apply to this Work are set out in our licence referred to above.

Other than as permitted in any relevant BMJ Author's Self Archiving Policies, I confirm this Work has not been accepted for publication elsewhere, is not being considered for publication elsewhere and does not duplicate material already published. I confirm all authors consent to publication of this Work and authorise the granting of this licence.

For peer review only

Acceptability of low-flow oxygen and bubble CPAP treatments among CPAP IMPACT caregivers in Malawi

Kristen L. Sessions[1,2], Laura M. Ruegsegger[1], Tisungane Mvalo[1,3], Davie Kondowe[1], Mercy Tsidya[1], Mina C. Hosseinipour[1,4], Norman Lufesi[5], Michelle Eckerle[6,7], Andrew Smith[8], Eric D. McCollum[9,10]

[1] University of North Carolina Project Malawi, Lilongwe, Malawi

[2] Department of Pediatrics, Lurie Children's Hospital of Northwestern University, Chicago, IL, USA

[3] Department of Pediatrics, School of Medicine, University of North Carolina at Chapel Hill, Chapel Hill, NC, USA.

[4] Division of Infectious Disease, University of North Carolina at Chapel Hill, Chapel Hill, NC, USA

[5] Malawi Ministry of Heath, Lilongwe, Malawi

[6] Division of Emergency Medicine, Cincinnati Children's Hospital Medical Center, Cincinnati, Ohio, USA

[7] University of Cincinnati College of Medicine, Cincinnati, Ohio, USA

[8] Pediatric Critical Care Medicine, University of Utah, Salt Lake City, Utah, USA

[9] Eudowood Division of Pediatric Respiratory Sciences, Department of Pediatrics, School of Medicine, Johns Hopkins University, Baltimore, Maryland, United States

[10] Health Systems Program, Department of International Health, Bloomberg School of Public Health, Johns Hopkins University, Baltimore, Maryland, United States

For peer review only

Corresponding authors:

Kristen L. Sessions, BS

University of North Carolina Project Malawi, Lilongwe, Malawi

Lurie Children's Hospital of Northwestern University, Chicago, IL

225 E Chicago Ave, Chicago, IL, 60611, USA

Phone: 312-227-3220

Email: ksessions@luriechildrens.org

Dr. Eric D. McCollum, MD

Director, Johns Hopkins University Global Program in Pediatric Respiratory Sciences

Assistant Professor of Pediatrics, Eudowood Division of Pediatric Respiratory Sciences, Johns Hopkins School of Medicine

Department of International Health, Johns Hopkins Bloomberg School of Public Health

200 North Wolfe Street

Baltimore, 21287, United States

Phone: 410-955-2035

Fax: 410-955-1030

Email: emccoll3@jhmi.edu

Word Count: 3,647

For peer review only

ABSTRACT

Objective: To determine the acceptability of bubble continuous positive airway pressure (bCPAP) and low-flow oxygen among mothers of children who had received either therapy.

Setting: A district hospital in Salima, Malawi

Participants: We conducted eight focus group discussions (FGD) with a total of 54 participants. Eligible participants were mothers of children 1-59 months of age with severe pneumonia and a co-morbidity (HIV-infection, HIV-exposure, malnutrition, or hypoxemia) who had been enrolled in a randomized clinical trial comparing low-flow oxygen and bCPAP treatments (ClinicalTrials.gov NCT02484183).

Primary and Secondary Outcome measures: FGDs assessed mothers' attitudes and feelings towards oxygen and bCPAP before and after therapy along with general community perceptions of respiratory therapies. Data was analyzed using inductive thematic analysis to assess themes and subthemes of the transcripts.

Results: Community perceptions of oxygen and bCPAP were widely negative. Mothers recounted that they are told that "oxygen kills babies." They are often fearful of allowing their child to receive oxygen therapy and will delay treatment or seek alternative therapies. Mothers report limiting oxygen and bCPAP by intermittently removing the nasal cannulas or mask. After oxygen or bCPAP treatment, regardless of patient outcome, mothers were supportive of the treatment their child received and would recommend it to other mothers.

Conclusion: There are significant community misconceptions around oxygen and bCPAP causing mothers to be fearful of either treatment. In order for low-flow oxygen treatment and bCPAP implementation to be effective, widespread community education is necessary.

For peer review only

Key words: Focus Groups, Developing Countries, Respiratory Tract Diseases, Child, Infant, Continuous Positive Airway Pressure, Hypoxia

ARTICLE SUMMARY

Strengths and limitations of the study

- Focus group discussions included mothers of surviving or deceased children who had severe pneumonia and received either oxygen or bCPAP therapy.
- This study was part of a larger randomized control trial, CPAP IMPACT, which sought to compare hospital outcomes between bCPAP and low-flow oxygen recipients with severe clinical pneumonia.
- Groups were facilitated by a trained Malawian facilitator and data was collected until thematic saturation was reached, suggesting sufficient sample size for addressing acceptability.
- This study is limited in that it may not be representative of other community beliefs regarding healthcare and mothers who did not present to the hospital or declined focus group participation were not included. Additionally, all focus groups were conducted post-intervention.

For peer review only

INTRODUCTION

Pneumonia remains a leading cause of death in young children worldwide with 50% of mortality occurring in sub-Saharan Africa.[1] Current standard of care for children in low-income African countries, such as Malawi, with low blood oxygen saturation – hypoxemia – is low-flow oxygen. Malawi initiated the Child Lung Health Program in October 2000, which installed oxygen concentrators in each district hospital and trained health care workers in proper indications for oxygen use.[2 3] Despite these efforts, mortality remains high and there may be a need for advanced treatment modalities to further decrease child mortality from pneumonia.

Bubble continuous positive airway pressure (bCPAP), a form of non-invasive ventilation that provides continuous positive end expiratory pressure (PEEP) to spontaneously breathing patients, has been shown to reduce mortality in neonates with respiratory distress.[4 5] However, evidence of effectiveness of bCPAP among older children is less conclusive.[6] Although the effectiveness of bCPAP in this age group is uncertain, implementation is ongoing in many low and middle income countries including Malawi, Kenya, Ghana, Honduras, and Malaysia.[6-10]

For an existing technology like oxygen and a new technology such as bCPAP to be most effective, they must be feasible to administer by healthcare personnel and acceptable to caregivers so that treatment adherence is optimized. Previous studies have demonstrated that bCPAP use on infants can be stressful and frightening to mothers.[11-13] In-depth interviews found mothers of neonates receiving bCPAP in Malawi were anxious and fearful, most indicating that they received inadequate information about the treatment.[11] Commonly quoted sources of stress include tubing, decreased ability for physical contact, and limited parent-child interactions.[12 14] In

For peer review only

this study, we sought to determine if mothers of children 1-59 months of age with pneumonia found oxygen and bCPAP acceptable. We also sought to identify community knowledge and feelings toward various treatment options for pneumonia.

METHODS

Study Design

In February and March of 2018, we conducted eight focus group discussions (FGD). Groups consisted of 6-10 mothers of children 1-59 months old who had previously received care for pneumonia as part of the randomized control trial CPAP IMPACT (Improving Mortality for Pneumonia in African Children Trial, clinicaltrial.gov NCT02484183), which sought to compare hospital outcomes between bCPAP and low-flow oxygen recipients with severe clinical pneumonia.[15] Focus groups were stratified by the trial's oxygen or bCPAP study groups and also whether the child survived or died in the hospital.

FGDs were held in a private room at Salima District Hospital (SDH) and were conducted in Chichewa, the local language, by a trained facilitator. Information about perceptions, education, and reactions toward bCPAP or oxygen were elicited. Each session lasted for about one hour and was audio recorded along with detailed notes taken by an independent note taker. A debriefing session with the study team took place after each session.

Sampling

Mothers were chosen by opportunistic and convenience sampling by contacting mothers whose child had been enrolled in CPAP IMPACT beginning with the most recent discharges. All

For peer review only

mothers whose child had been enrolled in CPAP IMPACT were eligible to participate in a FGD. Emancipated minors were allowed to participate. They were contacted via phone and invited to return to the hospital to participate. Contact information was missing for more mothers whose child died than whose child survived. However, among mothers who were successfully reached, no-show rates for FGDs were similar between mothers whose child survived and those who died. Mothers received a travel stipend and snack for participation in the FGD.

Data Management and Analytics

Focus group discussions were audio recorded, transcribed, translated from Chichewa to English, and then back translated to ensure meaning was preserved. Chichewa is a gender-neutral language so "they" or "them" were used throughout translations. Data was managed using Nvivo 11 QRS software. Transcripts were read in full to determine tone and scope of information. They were each coded by two independent reviewers (KS and LR) for themes and subthemes using inductive thematic analysis and a framework analysis approach.[16-18] Initial codes were developed *a priori* based on the interview guide but an iterative coding process was implemented to allow for flexibility and responsiveness to discussion results (Appendix 1). Themes, subthemes, and connections between concepts were noted and discussed with the team.

Ethical Considerations

This study was approved by both the National Health Research Science Committee of Malawi (1325) and the Institutional Review Board of John Hopkin's University (IRB00199008). Written consent was obtained in Chichewa from all participants. Consents were read aloud to participants who were unable to read. Data was de-identified prior to analysis.

For peer review only

Patient and Public Involvement.

It was not possible to involve patients or the public in the design, or conduct, or reporting, or dissemination of our research.

RESULTS

Demographics

A total of 54 participants took part in one of eight focus groups. Participants were an average of 26.7 years old and had 2.7 children (Table 1). Most participants (n=43; 79.6%) had a low socio-economic status as evidenced by living in a home without a metal roof and 74.1% (n=40) did not complete primary school.

Table 1: Demographics of mothers who participated in a focus group discussion

	Total (n= 54)	bCPAP (n= 25)	Oxygen (n= 29)	p value
Age in years, mean (SD)	26.7 (6.3)	26.5 (5.8)	26.9 (6.7)	0.7952
Number of children, mean (SD)	2.7 (1.5)	2.7 (1.4)	2.6 (1.7)	0.8877
Straw roof, n (%)*	43 (79.6)	21 (84.0)	22 (75.9)	0.9635
Child died during study, n (%)	11 (20.4)	5 (20.0)	6 (20.7)	0.8580
Completed primary school or higher education, n (%)	14 (25.9)	6 (24.0)	8 (27.6)	0.7791
Previous exposure to someone who was given oxygen therapy, n (%)	7 (13.0)	4 (16.0)	3 (10.3)	0.7833

*straw roof compared to a metal sheet roof was used as a marker of socioeconomic status

Major Themes

Throughout the focus groups, multiple themes arose including widespread negative beliefs about oxygen and bCPAP in the community, anxiety and fear surrounding care before initiation of treatment, and a shift in beliefs to a more positive opinion of treatment regardless of whether

For peer review only

their child lived or died (Table 2). Additionally, participants were able to identify potential benefits, harms, and alternatives to both oxygen and bCPAP.

Knowledge and feelings about oxygen and bCPAP prior to treatment

Knowledge of both oxygen and bCPAP was low within the participants' communities. Before participation in this study, only 13% (7/54) of participants personally knew someone who had received oxygen therapy. Despite limited personal exposure to oxygen, all mothers had heard of oxygen from members of their communities. Almost all information participants received from the community was negative. Eighty-three percent of mothers (45/54) explicitly, and without prompting, mentioned hearing negative opinions of supplemental oxygen therapy in the community, many using the phrasing that "oxygen kills" (Table 2).

> "At the village, people say that oxygen kills babies. So, when they told me that my baby will be on oxygen, I was very much worried about it." (oxygen, age 19, child survived)

They were also told to refuse therapy. One mother described her experience by saying,

> "People back in the village tell you to refuse the oxygen when you come to the hospital because they say a child who is put on an oxygen machine dies easily." (oxygen, age 33, child survived)

Not only are negative things said in the community, but also within the hospital. Mothers often reported being told to remove the equipment temporarily or permanently by other caregivers and this led to several self-reports of interrupting care.

> "When you are in that room, people stop what they were doing and start looking at you. Some [mothers] say 'I thought they are supposed to be removing it when the doctor is not there?'… They say because [bCPAP] kills." (bCPAP, age 33, child survived)

For peer review only - http://bmjopen.bmj.com/site/about/guidelines.xhtml

Some patients reported observing mothers abscond with their child. One mother also reported having her child taken off therapy by a relative.

> "One mother ran away with her sick child… She was afraid that oxygen kills. People were telling her that oxygen kills." (oxygen, age 24, child survived)

The knowledge about bCPAP was significantly lower; most reported they had never heard of bCPAP before being educated by the study staff at the hospital. However, those who had heard of bCPAP reported similar negative concern that it can worsen the child's condition or kill the child.

> "People in the community say that when a child is on bCPAP, they do not feed because the nose is covered and the child cannot swallow and they say it doesn't take long before the child dies" (bCPAP, age 35, child died)

In both groups, community beliefs led mothers to feel anxious and fearful prior to treatment initiation. Additional sources of fear included lack of familiarity with the machine and the severity of illness of the other children receiving treatment.

> "I was scared because it was my first time to see that thing and I was very worried... When I was entering the room, I found another child dying. So my feeling was that my child was going to die because that is the room where children die." (bCPAP, age 17, child survived)

When one mother was asked about the cause of her anxiety, she responded, "Primarily it was because of what people were saying. But since I had never seen this before, my worry multiplied." (oxygen, age 24, child survived)

For peer review only

Some women did not endorse anxiety around treatment. Instead, women in both groups referred to religious faith and trust in the medical providers as sources of comfort before treatment.

> “I also did not mind what people were saying in the community… I said ‘My child will receive this treatment; whatever happens to them; it is the will of God.’” (bCPAP, age 35, child died)

For peer review only

Table 2: Major themes that emerged

Community Knowledge	Overall low knowledge, with less knowledge about bCPAP compared to oxygen	"We are coming from the communities where we have no knowledge about this [bCPAP]."
	Negative thoughts about supplemental oxygen: "Oxygen kills"	"In our community, we are told that when someone is on oxygen they die so quickly because oxygen increases the death pace."
Feelings before Treatment	Almost all very anxious due to things said in the community or the appearance of the oxygen cannula or bCPAP machines and facemasks	"People say oxygen kills and this makes people feel afraid of the treatment. Someone ran away because she was afraid of the oxygen "
Alternative Treatments	Traditional medicine includes use of herbs and "traditional medicine tattoos"	"My granny tried to help my baby using herbs …She used some to rub herbs against their ribs, and some she made them drink, but it never worked out."
	Baby should be kept warm and environment clean	"The main cause of pneumonia is cold so if the child is kept warm it helps in that no coldness can penetrate them again."
	Injections or paracetamol can be given at health centers	"When a child has suffered from pneumonia and you go to the hospital, the doctors give the child injection."
Feelings after Treatment	Caregivers remained supportive of both oxygen and bCPAP regardless of child's outcome	"Oxygen helps but where the condition is not reversible, it does not. It takes someone to be lucky but it is helpful."
	Frequently stated that they would encourage others to seek treatment	"I am capable of encouraging them... I tell them that if a child has pneumonia, take them to the hospital and if they want to give them bCPAP, accept it."
Benefits to treatment	Helps the child breathe, allows child to feed and become more active, and aids in the child's survival	"I felt good because my child was able to feed while they had the oxygen tubes in the nose."
Harms to Treatment	Majority had no concerns, though some worried about bruising, rashes, and nasal injury and bCPAP machine malfunctions	"I don't think oxygen therapy can harm a child in any way because the oxygen that is found in the machine is the same that we breathe normally"
Education Timing	Majority prefer information to be given before treatment	"It is not practical to just put the child on bCPAP before giving information to the mother… people say that this thing kills and if that can happen, the mother can be so afraid and maybe she can run away."
	Desire for community sensitization	"I feel it is better to sensitize the people in the communities prior to their visits to the clinic because the people will have the information already."

For peer review only

Alternative treatments for the child's illness

Participants provided several examples of pneumonia treatments that have been used in the community. Many referred to traditional medicine techniques which consist of using herbs to burn and inhale the smoke, to rub on the baby's ribs, or to make drinks. Traditional healers may make "traditional medicine tattoos" in which herbs are placed beneath the child's skin via cuts or "tie the baby" which refers to making a necklace with a bag of grass and traditional medicine and placing it around the baby's neck or waist.

> "When we are at the village, we are told that the best way is to go to the traditional doctors. The traditional doctor makes traditional medicine tattoos and they also provide other medicines to drink so that the baby should be urinating frequently. After those medicines, they provide other medicines as well that they tie the baby. They say that when the baby is tied, it helps pneumonia to stop." (oxygen group, age 21, child survived)

While multiple alternative medicine techniques were endorsed, the majority of mothers who participated in the FGDs agreed that the best treatment is to seek care at a health center. Mothers who had tried traditional medicine before commented that they did not see improvement.

> "It is just very unfortunate that people go for traditional medicine but the best thing to do is to rush to the hospital." (bCPAP group, age 26, child survived)

Other proposed treatments for pneumonia included keeping the baby warm and medications such as antibiotics and acetaminophen.

Beliefs and feelings after treatment with oxygen or bCPAP

For peer review only - http://bmjopen.bmj.com/site/about/guidelines.xhtml

After treatment, the mothers were supportive of their care regardless of her child's health outcome and perceived multiple benefits of both treatments. Mothers whose child survived on oxygen or bCPAP expressed happiness and gratitude and they would recommend it to others.

> "My child was coming here when they were gasping...bCPAP recovered the child... It saved the life of my child and I will never forget. I will proclaim the benefits of bCPAP wherever I will go and I will encourage people to go for this treatment whenever they are offered." (bCPAP, age 35, child survived)

Mothers who had a child die while on therapy were still accepting of treatment. They often referred to other children who improved as evidence for its effectiveness.

> "Eventually my child died, not because they were on bCPAP but because it was time for them to die. So I cannot say that bCPAP kills just because my child did not survive; it is helpful because I saw other children surviving but I was just unfortunate that my child died." (bCPAP, age 30, child died)

Perceived benefits and harms of oxygen and bCPAP

The most commonly discussed benefit was that the machines help children breathe. Feeding was also often used as a marker of health with mothers referencing the ability to feed again as a benefit to both oxygen and bCPAP treatments. Once on treatment, their child became more active. Overall, mothers claimed that the machine helped their child to survive.

> "Oxygen is helpful. Even when a child is critically ill but when they put on oxygen the child is able to feed." (oxygen, age 38, child died)

> "I feel [bCPAP] increases the oxygen levels and help the child to breathe properly. If the child was not able to feed, they start feeding." (bCPAP, age 30, child died)

For peer review only - http://bmjopen.bmj.com/site/about/guidelines.xhtml

A majority of the mothers had no concerns about treatment. Some mothers were concerned about bruising, rashes, or nasal injury. Other concerns included machine malfunctions or electricity black outs, which are common in Malawi.

> "My only observation was that after receiving the treatment, my child had red skin meaning that the belt had caused some friction on the skin." (bCPAP, age 35, child survived)

Education about oxygen and bCPAP

Given the low community knowledge about oxygen and bCPAP, mothers were asked about the best time to be given information about treatment. The majority of mothers preferred information about the treatment to be given before any medical care was provided to the child to address any concerns or allow the mother to decline treatment.

> "We are coming from the communities where we have no knowledge about this thing. What people talk about this thing is different from what is on the ground. So it is better to give the information about it before giving treatment so that people are well sensitized on how it works in order to deal with their fears they had from the community." (bCPAP, age 35, child died)

However, several felt it was more important to start the child on treatment first then provide information to the caregiver. This was often the case when they felt the child was very sick and treatment should not be delayed.

> "[With] how serious the child is; it is better to start treating the child before giving the information so that the child should get better." (bCPAP, age 26, child survived)

For peer review only - http://bmjopen.bmj.com/site/about/guidelines.xhtml

"If you have gone to the hospital sometimes the severity of the illnesses differs …I feel that the proper thing would be to start treating the child first with the oxygen therapy then they can start giving you the information." (oxygen group, age 24, child survived)

Another common theme was the desire for education in the communities. Mothers recognized a need for community sensitization to feel more comfortable about coming to the hospital.

"I would love if this information is given to us before the child gets sick so that when we come here, it should not be a strange thing to us. They can use the health surveillance assistants to sensitize the communities so that by the time the child will be sick, when we come here, we should not waste time getting the information but instead the child should start treatment right away." (bCPAP, age 26, child survived)

DISCUSSION

We found that many communities have negative beliefs and fears of supplement oxygen. In the communities, many women have been told that "oxygen kills" and offered alternative traditional medicine. They have been told to remove the oxygen therapy, which causes interruptions in treatment. Most caregivers were experiencing supplemental oxygen for the first time, and they reported feeling anxious mostly due to the community input. Regardless of the randomization group and outcome, after receiving treatment the caregivers reported satisfaction with the treatment and would even encourage others to go to the hospital for care. Mothers of children who died referenced temporary improvement in their child's clinical status or seeing other children improve with treatment as evidence for its effectiveness. This suggests that education and exposure to the treatments, even in the setting of a poor outcome, can be effective.

For peer review only - http://bmjopen.bmj.com/site/about/guidelines.xhtml

Many participants saw the benefits of oxygen and bCPAP care, and little saw harm aside from bruising or other marks from wearing the mask. Most mothers prefer information about treatment to be given before it is started and believe that the community would benefit from sensitization.

Oxygen therapy has been standard of care at SDH for children with severe pneumonia since Malawi implemented oxygen throughout the country beginning in 2000[3]. Despite availability for 18 years, mothers still reported widespread negative beliefs in their communities. bCPAP had only been available at SDH through CPAP IMPACT for 2 years prior to these focus group sessions. Even though it was less familiar to mothers, those who had heard of it expressed similar fears and beliefs. Mothers of neonates receiving bCPAP in a NICU in Malawi expressed similar fears and anxieties.[11] Both studies demonstrated satisfaction with the treatment after initiation suggesting that exposure and education can reduce anxiety in mothers of children requiring respiratory support.

The perception that oxygen can kill children could lead mothers to delay seeking care and to seek other treatment options in their communities. Caregivers reported seeing traditional healers or trying home treatments before coming to the hospital. It is unclear how long caregivers delayed seeking more formal healthcare while under the care of traditional healers. They also reported feeling desperate when they arrived at the hospital and willing to accept any treatment given the condition of their child. If only the sickest children receive oxygen, mortality will likely remain high, which may continue to propagate negative beliefs among the community. Even once arriving at the hospital, we found mothers reported continuing to hear negative opinions from other caregivers and were deterred from accepting or continuing treatment. The intermittent

removal of the mask or nasal prongs by caregivers during oxygen and bCPAP treatments could interfere with treatment effectiveness and lead to poorer than expected outcomes. Caregivers implied that removal of oxygen and bCPAP treatments occurred when healthcare providers were not present, perhaps while caring for other children or at night when staffing levels were lower. While the frequency of removal was unclear, its occurrence is nevertheless alarming. These findings suggest that in the absence of more intensive and widespread community sensitization the use of oxygen and bCPAP in Malawi may be more effective in units with higher provider to parent ratios to facilitate closer monitoring of parental interactions with their children, rather than in general pediatric wards as was done in CPAP IMPACT. District hospitals in Malawi do not have high dependency or intensive care units.

Oxygen support has been shown to be critically important in the care of children with respiratory distress and is included in the WHO list of essential medicines. For oxygen to have its full benefit and for implementation of newer technologies such as bCPAP to be effective, there is a clear need for community education. Caregivers displayed a strong support for both oxygen and bCPAP following treatment, even if their child died. This suggests that with exposure and education both can be considered acceptable therapies among caregivers. Caregivers in multiple groups expressed a desire for information in their communities before their children were sick. Community health workers (CHWs) have been shown to be effective in both community management of some diseases and preventative measures.[20] Community health workers delivering preventative care in LMICs have been found to be effective in community education.[21] Similar efforts may be effective in increasing education about respiratory support technologies.

Throughout focus group discussions, mothers referred to feeding as a surrogate marker for health. They described needing to bring the child to the hospital because they had stopped feeding. The ability of the child to eat was often cited as a benefit of therapy and a way of seeing improvement. There have been strong efforts in Malawi to encourage exclusive breast feeding and their child's ability to eat was clearly important to mothers. This may potentially help explain some of the findings of CPAP IMPACT, which showed increased risk mortality in the bCPAP group, compared to the oxygen group.[15] The authors speculated that these findings could be in part due to a higher risk of aspiration from oral feeding while receiving positive pressure ventilation from bCPAP, compared to low flow oxygen. The use of feeding as a marker of health status supports the concern that mothers may have continued to feed orally despite the child's decreased respiratory status and the provider's recommendation against oral feeding.

There are several notable limitations. First, the views of study participants may not be representative of all views. Mothers who participated in a group had already consented to having their child receive therapy and all focus groups were conducted post-intervention. Groups did not include mothers who never presented to the hospital. This population is harder to reach and would require resources beyond the capacity of this study. However, participants were able to clearly talk about community views and opinions they had heard from their peers and the sample size of women included was sufficient to achieve thematic saturation. Second, the beliefs of participants may not be generalizable to other contexts and oxygen use and bCPAP implementation in other settings will need to consider unique culture viewpoints. Fewer women with deceased children participated in the focus groups. However, thematic saturation was

achieved, and this group's views did not differ significantly from caregivers whose children survived. Lastly, in this setting, mothers may be compelled to provide answers that they believe are desired by the research team. We attempted to avoid this by explaining to mothers that all opinions were open and by choosing a facilitator who was independent of clinical care and had never met or interacted with mothers prior. Focus group meetings took place outside the hospital ward in a private room and only Malawians were present to encourage honesty and openness among participants.

CONCLUSION

In Malawi there are widespread negative beliefs about oxygen and bCPAP therapies with community members often citing that "oxygen kills babies." These beliefs may lead to delays in care for children with respiratory disease and may also interrupt care within the hospital. Mothers reported feeling anxious about allowing their child to receive treatment. Widespread community sensitization is needed to educate communities and improve acceptance of oxygen and other respiratory therapies. Additionally, these results suggest that the role of feeding and its relationship to the effectiveness of respiratory therapies in Malawi needs further evaluation.

DECLARATIONS

Funding

KS received funding for this work through a Doris Duke Charitable Foundation grant (2016177) supporting the Doris Duke International Clinical Research Fellows Program at the University of North Carolina Chapel Hill. The Bill and Melinda Gates Foundation (OPP1123419),

For peer review only - http://bmjopen.bmj.com/site/about/guidelines.xhtml

International AIDS Society (141022), and Health Empowering Humanity provided funding for CPAP IMPACT.

Conflicts of Interests

The authors declare that they have no conflicts of interests.

Author Statement

KS, LR, TM, and EDM participated in study design, data collection, and statistical analysis and were major contributors to manuscript writing. MT participated in FGD guide preparation, conducted the FGDs, completed translation and transcription, and contributed to analysis and manuscript preparation. DK contributed to data collection and design and implementation of CPAP IMPACT. MH, NL, AS, ME, and EDM contributed to study design and implementation of CPAP IMPACT. EDM was the principle investigator. All authors contributed to manuscript preparation and approved the final manuscript.

Acknowledgments

We thank caregivers and their children for participating in this trial, the Malawi Ministry of Health, and the CPAP-IMPACT study team for their contributions to this study.

Data Sharing

De-identified transcripts of FGDs are available on request from corresponding authors.

For peer review only

References

1. Troeger C, Forouzanfar M, Rao PC, et al. Estimates of the global, regional, and national morbidity, mortality, and aetiologies of lower respiratory tract infections in 195 countries: a systematic analysis for the Global Burden of Disease Study 2015. *The Lancet Infectious Diseases* 2017;17(11):1133-61.
2. Enarson PM, Gie RP, Mwansambo CC, et al. Reducing deaths from severe pneumonia in children in Malawi by improving delivery of pneumonia case management. *PloS one* 2014;9(7):e102955-e55. doi: 10.1371/journal.pone.0102955
3. Enarson P, La Vincente S, Gie R, et al. Implementation of an oxygen concentrator system in district hospital paediatric wards throughout Malawi. *Bulletin of the World Health Organization* 2008;86(5):344-48.
4. Nowadzky T, Pantoja A, Britton JR. Bubble continuous positive airway pressure, a potentially better practice, reduces the use of mechanical ventilation among very low birth weight infants with respiratory distress syndrome. *Pediatrics* 2009;123(6):1534-40. doi: 10.1542/peds.2008-1279 [published Online First: 2009/06/02]
5. DiBlasi RM. Nasal continuous positive airway pressure (CPAP) for the respiratory care of the newborn infant. *Respiratory care* 2009;54(9):1209-35.
6. Wilson PT, Morris MC, Biagas KV, et al. A randomized clinical trial evaluating nasal continuous positive airway pressure for acute respiratory distress in a developing country. *The Journal of pediatrics* 2013;162(5):988-92.
7. Walk J, Dinga P, Banda C, et al. Non-invasive ventilation with bubble CPAP is feasible and improves respiratory physiology in hospitalised Malawian children with acute respiratory failure. *Paediatrics and international child health* 2016;36(1):28-33. doi: 10.1179/2046905514y.0000000166 [published Online First: 2014/12/02]
8. Machen HE, Mwanza ZV, Brown JK, et al. Outcomes of patients with respiratory distress treated with bubble CPAP on a pediatric ward in Malawi. *Journal of tropical pediatrics* 2015;61(6):421-27.
9. Lum LC, Abdel-Latif ME, de Bruyne JA, et al. Noninvasive ventilation in a tertiary pediatric intensive care unit in a middle-income country. *Pediatric Critical Care Medicine* 2011;12(1):e7-e13.
10. Wilson PT, Benckert MM, Moresky RT, et al. Development and Implementation of a Training-of-Trainers Program for Continuous Positive Airway Pressure in Neonatal and Pediatric Patients in Five Low-and Middle-Income Countries. *Journal of tropical pediatrics* 2017;63(5):358-64.
11. Gondwe MJ, Gombachika B, Majamanda MD. Experiences of caregivers of infants who have been on bubble continuous positive airway pressure at Queen Elizabeth Central Hospital, Malawi: A descriptive qualitative study. *Malawi Medical Journal* 2017;29(1):5-10.
12. Peeler A, Fulbrook P, Kildea S. The experiences of parents and nurses of hospitalised infants requiring oxygen therapy for severe bronchiolitis: A phenomenological study. *Journal of Child Health Care* 2015;19(2):216-28.
13. Foster J, Bidewell J, Buckmaster A, et al. Parental stress and satisfaction in the non-tertiary special care nursery. *Journal of advanced nursing* 2008;61(5):522-30.
14. Cervantes AC, Feeley N, Lariviere J. The experience of mothers whose very low-birth-weight infant requires the delivery of supplemental oxygen in the neonatal intensive care unit. *Advances in Neonatal Care* 2011;11(1):54-61.

For peer review only

15. McCollum ED, Mvalo T, Eckerle M, Smith AG, Kondowe D, Makonokaya D, Vaidya D, Billioux V, Chalira A, Lufesi N, Mofolo I, Hosseinipour M. Bubble continuous positive airway pressure for children with high-risk conditions and severe pneumonia in Malawi: an open label randomized controlled trial. Lancet Resp Med. 2019; In press
16. Ritchie J, Spencer L. Qualitative data analysis for applied policy research. *The qualitative researcher's companion* 2002;573(2002):305-29.
17. Patton MQ. Qualitative research: Wiley Online Library 2005.
18. Strauss A, Corbin JM. Basics of qualitative research: Grounded theory procedures and techniques: Sage Publications, Inc 1990.
19. World Health Organization Model List of Essential Medicines, 21st List, 2019. Geneva: World Health Organization; 2019. Licence: CC BY-NC-SA 3.0 IGO.
20. Haines A, Sanders D, Lehmann U, et al. Achieving child survival goals: potential contribution of community health workers. *The Lancet* 2007;369(9579):2121-31.
21. Gilmore B, McAuliffe E. Effectiveness of community health workers delivering preventive interventions for maternal and child health in low-and middle-income countries: a systematic review. *BMC public health* 2013;13(1):847.

For peer review only

Appendix 1: Example facilitator guide for focus group discussions with mothers whose child was on oxygen

Thank you all for joining us for this discussion. You have been invited to participate in this discussion because your child participated in a study where they were randomized to receive either bubble CPAP or oxygen therapy for severe pneumonia. We wanted to better understand your thoughts on treatment for pneumonia in young children and your experiences with oxygen.

1. Had you seen or heard about oxygen therapy before your child was hospitalized at Salima District Hospital?
    a. If so, please discuss what you had seen or heard.
    b. Had you heard about bubble CPAP, the other treatment used in the study?
2. What is your understanding of what oxygen therapy is?
    a. Where did you learn about oxygen therapy?
        i. PROBE: Can you identify who at the hospital provided you with information or was able to answer your questions?
    b. Do you feel like you received enough information or teaching about oxygen therapy?
        i. When did this teaching occur?
        ii. PROBE: Did you learn about oxygen therapy before or after your child was started on treatment?
3. How did you feel about your child receiving oxygen therapy at the beginning?
    a. What concerns did you have?
        i. PROBE: Did you hope your child would be in the oxygen therapy or bubble CPAP arm of the study? Why?

For peer review only

  b. Did your feelings about oxygen therapy change during the hospitalization?
     i. PROBE: Can you describe what it felt like to see your child on oxygen therapy?

4. What are the benefits of oxygen therapy for your child?
   a. PROBE: Can someone give me an example of a way that oxygen therapy may benefit a child with pneumonia?
5. Do you have any concerns that oxygen therapy can harm your child?
   a. If so, please explain.
   b. Do you think others in the community would have concerns about oxygen therapy?
6. What are other types of treatments for pneumonia?
   a. Are they better or worse? Why?

Thank you for your participation in this discussion today. This information will help us design future educational materials to help providers talk to mothers and caregivers about bubble CPAP and treatment for severe pneumonia. Please let us know if you have any questions. *[Clarify any misconceptions if any came up during the discussion.]*

For peer review only

Reporting checklist for qualitative study.

Based on the SRQR guidelines.

Instructions to authors

Complete this checklist by entering the page numbers from your manuscript where readers will find each of the items listed below.

Your article may not currently address all the items on the checklist. Please modify your text to include the missing information. If you are certain that an item does not apply, please write "n/a" and provide a short explanation.

Upload your completed checklist as an extra file when you submit to a journal.

In your methods section, say that you used the SRQRreporting guidelines, and cite them as:

O'Brien BC, Harris IB, Beckman TJ, Reed DA, Cook DA. Standards for reporting qualitative research: a synthesis of recommendations. Acad Med. 2014;89(9):1245-1251.

		Reporting Item	Page Number
**Title**			
	#1	Concise description of the nature and topic of the study identifying the study as qualitative or indicating the approach (e.g. ethnography, grounded theory) or data collection methods (e.g. interview, focus group) is recommended	1
**Abstract**			
	#2	Summary of the key elements of the study using the abstract format of the intended publication; typically includes background, purpose, methods, results and conclusions	3
**Introduction**			
Problem formulation	#3	Description and signifcance of the problem / phenomenon studied: review of relevant theory and empirical work; problem statement	5

For peer review only

Purpose or research question	#4	Purpose of the study and specific objectives or questions	5
**Methods**			
Qualitative approach and research paradigm	#5	Qualitative approach (e.g. ethnography, grounded theory, case study, phenomenolgy, narrative research) and guiding theory if appropriate; identifying the research paradigm (e.g. postpositivist, constructivist / interpretivist) is also recommended; rationale. The rationale should briefly discuss the justification for choosing that theory, approach, method or technique rather than other options available; the assumptions and limitations implicit in those choices and how those choices influence study conclusions and transferability. As appropriate the rationale for several items might be discussed together.	6
Researcher characteristics and reflexivity	#6	Researchers' characteristics that may influence the research, including personal attributes, qualifications / experience, relationship with participants, assumptions and / or presuppositions; potential or actual interaction between researchers' characteristics and the research questions, approach, methods, results and / or transferability	6
Context	#7	Setting / site and salient contextual factors; rationale	6
Sampling strategy	#8	How and why research participants, documents, or events were selected; criteria for deciding when no further sampling was necessary (e.g. sampling saturation); rationale	6
Ethical issues pertaining to human subjects	#9	Documentation of approval by an appropriate ethics review board and participant consent, or explanation for lack thereof; other confidentiality and data security issues	7
Data collection methods	#10	Types of data collected; details of data collection procedures including (as appropriate) start and stop dates of data collection and analysis, iterative	7

For peer review only

		process, triangulation of sources / methods, and modification of procedures in response to evolving study findings; rationale	
Data collection instruments and technologies	#11	Description of instruments (e.g. interview guides, questionnaires) and devices (e.g. audio recorders) used for data collection; if / how the instruments(s) changed over the course of the study	7
Units of study	#12	Number and relevant characteristics of participants, documents, or events included in the study; level of participation (could be reported in results)	8
Data processing	#13	Methods for processing data prior to and during analysis, including transcription, data entry, data management and security, verification of data integrity, data coding, and anonymisation / deidentification of excerpts	7
Data analysis	#14	Process by which inferences, themes, etc. were identified and developed, including the researchers involved in data analysis; usually references a specific paradigm or approach; rationale	7
Techniques to enhance trustworthiness	#15	Techniques to enhance trustworthiness and credibility of data analysis (e.g. member checking, audit trail, triangulation); rationale	7
**Results/findings**			
Syntheses and interpretation	#16	Main findings (e.g. interpretations, inferences, and themes); might include development of a theory or model, or integration with prior research or theory	8
Links to empirical data	#17	Evidence (e.g. quotes, field notes, text excerpts, photographs) to substantiate analytic findings	8
**Discussion**			
Intergration with prior work, implications, transferability and contribution(s) to the field	#18	Short summary of main findings; explanation of how findings and conclusions connect to, support, elaborate on, or challenge conclusions of earlier scholarship; discussion of scope of application /	16

For peer review only

		generalizability; identification of unique contributions(s) to scholarship in a discipline or field	
Limitations	#19	Trustworthiness and limitations of findings	19
**Other**			
Conflicts of interest	#20	Potential sources of influence of perceived influence on study conduct and conclusions; how these were managed	21
Funding	#21	Sources of funding and other support; role of funders in data collection, interpretation and reporting	20

The SRQR checklist is distributed with permission of Wolters Kluwer © 2014 by the Association of American Medical Colleges. This checklist was completed on 24. September 2019 using https://www.goodreports.org/, a tool made by the EQUATOR Network in collaboration with Penelope.ai

For peer review only

BMJ Open

Focus group discussions on low-flow oxygen and bubble CPAP treatments among mothers of young children in Malawi: a CPAP IMPACT sub-study

Journal:	*BMJ Open*
Manuscript ID	bmjopen-2019-034545.R1
Article Type:	Original research
Date Submitted by the Author:	11-Mar-2020
Complete List of Authors:	Sessions, Kristen; McGaw Medical Center of Northwestern University, Pediatrics<br>Ruegsegger, Laura; University of North Carolina System, Project Malawi<br>Mvalo, Tisungane; University of North Carolina Project Malawi, ; University of North Carolina at Chapel Hill, Department of Pediatrics<br>Kondowe, Davie; University of North Carolina Project Malawi<br>Tsidya, Mercy; University of North Carolina Project Malawi<br>Hosseinipour, Mina C.; University of North Carolina Project Malawi<br>Lufesi, Norman; Ministry of Health, Acute Respiratory Infection Unit<br>Eckerle, Michelle; Cincinnati Children's Hospital Medical Center, Pediatrics<br>Smith, Andrew; University of Utah<br>McCollum, Eric ; Johns Hopkins School of Medicine
**Primary Subject Heading**:	Paediatrics
Secondary Subject Heading:	Global health, Qualitative research
Keywords:	focus groups, developing countries, respiratory tract diseases, Child, continuous positive airway pressure, hypoxia

SCHOLARONE™ Manuscripts

I, the Submitting Author has the right to grant and does grant on behalf of all authors of the Work (as defined in the below author licence), an exclusive licence and/or a non-exclusive licence for contributions from authors who are: i) UK Crown employees; ii) where BMJ has agreed a CC-BY licence shall apply, and/or iii) in accordance with the terms applicable for US Federal Government officers or employees acting as part of their official duties; on a worldwide, perpetual, irrevocable, royalty-free basis to BMJ Publishing Group Ltd ("BMJ") its licensees and where the relevant Journal is co-owned by BMJ to the co-owners of the Journal, to publish the Work in this journal and any other BMJ products and to exploit all rights, as set out in our licence.

The Submitting Author accepts and understands that any supply made under these terms is made by BMJ to the Submitting Author unless you are acting as an employee on behalf of your employer or a postgraduate student of an affiliated institution which is paying any applicable article publishing charge ("APC") for Open Access articles. Where the Submitting Author wishes to make the Work available on an Open Access basis (and intends to pay the relevant APC), the terms of reuse of such Open Access shall be governed by a Creative Commons licence – details of these licences and which Creative Commons licence will apply to this Work are set out in our licence referred to above.

Other than as permitted in any relevant BMJ Author's Self Archiving Policies, I confirm this Work has not been accepted for publication elsewhere, is not being considered for publication elsewhere and does not duplicate material already published. I confirm all authors consent to publication of this Work and authorise the granting of this licence.

For peer review only

Focus group discussions on low-flow oxygen and bubble CPAP treatments among mothers of young children in Malawi: a CPAP IMPACT sub-study

Kristen L. Sessions[1,2], Laura M. Ruegsegger[1], Tisungane Mvalo[1,3], Davie Kondowe[1], Mercy Tsidya[1], Mina C. Hosseinipour[1,4], Norman Lufesi[5], Michelle Eckerle[6,7], Andrew Smith[8], Eric D. McCollum[9,10]

[1] University of North Carolina Project Malawi, Lilongwe, Malawi

[2] Department of Pediatrics, Lurie Children's Hospital of Northwestern University, Chicago, IL, USA

[3] Department of Pediatrics, School of Medicine, University of North Carolina at Chapel Hill, Chapel Hill, NC, USA.

[4] Division of Infectious Disease, University of North Carolina at Chapel Hill, Chapel Hill, NC, USA

[5] Malawi Ministry of Heath, Lilongwe, Malawi

[6] Division of Emergency Medicine, Cincinnati Children's Hospital Medical Center, Cincinnati, Ohio, USA

[7] University of Cincinnati College of Medicine, Cincinnati, Ohio, USA

[8] Pediatric Critical Care Medicine, University of Utah, Salt Lake City, Utah, USA

[9] Eudowood Division of Pediatric Respiratory Sciences, Department of Pediatrics, School of Medicine, Johns Hopkins University, Baltimore, Maryland, United States

[10] Health Systems Program, Department of International Health, Bloomberg School of Public Health, Johns Hopkins University, Baltimore, Maryland, United States

For peer review only

Corresponding authors:

Kristen L. Sessions, BS

University of North Carolina Project Malawi, Lilongwe, Malawi

Lurie Children's Hospital of Northwestern University, Chicago, IL

225 E Chicago Ave, Chicago, IL, 60611, USA

Phone: 312-227-3220

Email: ksessions@luriechildrens.org

Dr. Eric D. McCollum, MD

Director, Johns Hopkins Global Program in Pediatric Respiratory Sciences

Assistant Professor of Pediatrics, Eudowood Division of Pediatric Respiratory Sciences, Johns Hopkins School of Medicine

Department of International Health, Johns Hopkins Bloomberg School of Public Health

200 North Wolfe Street

Baltimore, 21287, United States

Phone: 410-955-2035

Fax: 410-955-1030

Email: emccoll3@jhmi.edu

Word Count: 3,647

For peer review only

ABSTRACT

Objective: To determine the acceptability of bubble continuous positive airway pressure (bCPAP) and low-flow oxygen among mothers of children who had received either therapy.

Setting: A district hospital in Salima, Malawi

Participants: We conducted eight focus group discussions (FGD) with a total of 54 participants. Eligible participants were mothers of children 1-59 months of age with severe pneumonia and a co-morbidity (HIV-infection, HIV-exposure, malnutrition, or hypoxemia) who, with informed consent, had been enrolled in a randomized clinical trial, CPAP IMPACT, comparing low-flow oxygen and bCPAP treatments (ClinicalTrials.gov NCT02484183).

Primary and Secondary Outcome measures: FGDs assessed mothers' attitudes and feelings towards oxygen and bCPAP before and after therapy along with general community perceptions of respiratory therapies. Data was analyzed using inductive thematic analysis to assess themes and subthemes of the transcripts.

Results: Community perceptions of oxygen and bCPAP were widely negative. Mothers recounted that they are told that "oxygen kills babies." They are often fearful of allowing their child to receive oxygen therapy and will delay treatment or seek alternative therapies. Mothers report limiting oxygen and bCPAP by intermittently removing the nasal cannulas or mask. After oxygen or bCPAP treatment, regardless of patient outcome, mothers were supportive of the treatment their child received and would recommend it to other mothers.

Conclusion: There are significant community misconceptions around oxygen and bCPAP causing mothers to be fearful of either treatment. In order for low-flow oxygen treatment and bCPAP implementation to be effective, widespread community education is necessary.

For peer review only

Key words: Focus Groups, Developing Countries, Respiratory Tract Diseases, Child, Infant, Continuous Positive Airway Pressure, Hypoxia

ARTICLE SUMMARY

Strengths and limitations of the study

- Focus group discussions included mothers of surviving or deceased children who had severe pneumonia and received either oxygen or bCPAP therapy.
- This study was part of a larger randomized control trial, CPAP IMPACT, which sought to compare hospital outcomes between bCPAP and low-flow oxygen recipients with severe clinical pneumonia.
- Groups were facilitated by a trained Malawian facilitator and data was collected until thematic saturation was reached, suggesting sufficient sample size for addressing acceptability.
- This study is limited in that it may not be representative of other community beliefs regarding healthcare and mothers who did not present to the hospital or declined focus group participation were not included. Additionally, all focus groups were conducted post-intervention.

For peer review only

INTRODUCTION

Pneumonia remains a leading cause of death in young children worldwide with 50% of mortality occurring in sub-Saharan Africa.[1] Current standard of care for children in low-income African countries, such as Malawi, with low blood oxygen saturation – hypoxemia – is low-flow oxygen. Malawi initiated the Child Lung Health Program in October 2000, which installed oxygen concentrators in each district hospital and trained health care workers in proper indications for oxygen use.[2,3] Despite these efforts, mortality remains high and there may be a need for advanced treatment modalities to further decrease child mortality from pneumonia.

Bubble continuous positive airway pressure (bCPAP), a form of non-invasive ventilation that provides continuous positive end expiratory pressure (PEEP) to spontaneously breathing patients, has been shown to reduce mortality in neonates with respiratory distress.[4,5] However, evidence of effectiveness of bCPAP among older children is less conclusive.[6] Although the effectiveness of bCPAP in this age group is uncertain, implementation is ongoing in many low-income and middle-income countries including Malawi, Kenya, Ghana, Honduras, and Malaysia.[6-10]

For an existing technology like oxygen and a new technology such as bCPAP to be most effective, they must be feasible to administer by healthcare personnel and acceptable to caregivers so that treatment adherence is optimized. Previous studies have demonstrated that bCPAP use on infants can be stressful and frightening to mothers.[11-13] In-depth interviews found mothers of neonates receiving bCPAP in Malawi were anxious and fearful, most indicating that they received inadequate information about the treatment.[11] Commonly quoted sources of stress

For peer review only

include tubing, decreased ability for physical contact, and limited parent-child interactions.[12 14] In this study, we sought to determine if mothers of children 1-59 months of age with pneumonia found oxygen and bCPAP acceptable. We also sought to identify community knowledge and feelings toward various treatment options for pneumonia.

METHODS

Study Design

In February and March of 2018, we conducted eight focus group discussions (FGD). Groups consisted of 6-10 mothers of children 1-59 months old who had previously received care for pneumonia as part of the randomized controlled trial CPAP IMPACT (Improving Mortality for Pneumonia in African Children Trial, clinicaltrial.gov NCT02484183), which sought to compare hospital outcomes between bCPAP and low-flow oxygen recipients with severe clinical pneumonia who, based on World Health Organization (WHO) recommendations, would benefit from supplemental oxygen therapy.[15] Prior to enrollment trial participants underwent a process of informed consent, which reviewed and demonstrated bCPAP and oxygen care, bCPAP devices including masks, prongs, and tubing, low-flow oxygen nasal cannulas, study blood draws, and also expected routine hospital care related to both modalities not limited to nasogastric tube feeding, intravenous fluids, blood draws, and nasal suctioning. The benefits and risks of trial participation were also reviewed during consent. Participants were permitted to withdraw from the main trial at any point. Focus groups were stratified by the trial's oxygen or bCPAP study groups and also whether the child survived or died in the hospital.

FGDs were held in a private room at Salima District Hospital (SDH) and were conducted in Chichewa, the local language, by a trained facilitator. Information about perceptions, education, and reactions toward bCPAP or oxygen were elicited. Mothers were asked to discuss beliefs they had heard from their communities, information learned through the study consent process, and experiences during their child's treatment. Each session lasted for about one hour and was audio recorded along with detailed notes taken by an independent note taker. A debriefing session with the study team took place after each session.

Sampling

Mothers were chosen by opportunistic and convenience sampling by contacting mothers whose child had been enrolled in CPAP IMPACT beginning with the most recent discharges. All mothers whose child had been enrolled in CPAP IMPACT were eligible to participate in a FGD. Women who were aged 16 years old and above but had a child were allowed to participate in accordance with Malawi ethical research standards and laws. They were contacted via phone and invited to return to the hospital to participate. Contact information was missing for more mothers whose child died than whose child survived. However, among mothers who were successfully reached, no-show rates for FGDs were similar between mothers whose child survived and those who died. Mothers received a travel stipend and snack for participation in the FGD, which is research participation compensation in accordance with Malawi ethical standards.

Data Management and Analytics

Focus group discussions were audio recorded, transcribed, translated from Chichewa to English, and then back translated to ensure meaning was preserved. Chichewa is a gender-neutral

For peer review only

language so "they" or "them" were used throughout translations. Data was managed using Nvivo 11 QRS software. Transcripts were read in full to determine tone and scope of information. They were each coded by two independent reviewers (KS and LR) for themes and subthemes using inductive thematic analysis and a framework analysis approach.[16-18] Initial codes were developed *a priori* based on the interview guide but an iterative coding process was implemented to allow for flexibility and responsiveness to discussion results (Appendix 1). Themes, subthemes, and connections between concepts were noted and discussed with the team. Statistical analyses for quantitative variables used mean and standard deviation to describe normally distributed data and median and interquartile range to describe not normally distributed data. We compared means of normally distributed data using t tests and medians of not normally distributed data using the Wilcoxon-Mann-Whitney test. Quantitative analyses were conducted using Stata 15.1 (College Station, USA).

Ethical Considerations

This study was approved by both the National Health Research Science Committee of Malawi (1325) and the Institutional Review Board of John Hopkin's University (IRB00199008). Written consent was obtained in Chichewa from all participants. Consents were read aloud to participants who were unable to read. Data was de-identified prior to analysis.

Patient and Public Involvement.

Due to resource limitations, it was not possible to involve patients or the public in the trial design, or conduct, or reporting, or dissemination of our research. However, members of the

For peer review only

Salima Distric Hospital administration and Malawi Ministry of Health were involved in the design, conduct, reporting, and dissemination of this study.

RESULTS

Demographics

A total of 54 participants took part in one of eight focus groups. Table 1 reports the demographics of mothers who participated and indicates that the characteristics of mothers participating in this sub-study were balanced between trial arms. Participants were an average of 26.7 years old and had 2.7 children. Most participants (n=43; 79.6%) had a low socio-economic status as evidenced by living in a home without a metal roof and 74.1% (n=40) did not complete primary school.

Table 1: Demographics of mothers who participated in a focus group discussion

	Total (n= 54)	bCPAP (n= 25)	Oxygen (n= 29)	p value
Age of mothers in years, median (IQR)	26 (21, 32)	26 (21, 31)	25 (22, 32)	0.8755
Number of children, mean (SD)	2.7 (1.5)	2.7 (1.4)	2.6 (1.7)	0.8887
Age of participant children in months, median (IQR)	7.1 (3.1, 14.2)	6.7 (3.1, 12.1)	8 (4.8, 14.2)	0.6148
Child died during study, n (%)	11 (20.4)	5 (20.0)	6 (20.7)	0.8580
Straw roof, n (%)*	43 (79.6)	21 (84.0)	22 (75.9)	0.9635
Completed primary school or higher education, n (%)	14 (25.9)	6 (24.0)	8 (27.6)	0.7791
Previous exposure to someone who was given oxygen therapy, n (%)	7 (13.0)	4 (16.0)	3 (10.3)	0.7833

*straw roof compared to a metal sheet roof was used as a marker of socioeconomic status

Major Themes

For peer review only

Throughout the focus groups, multiple themes arose including widespread negative beliefs about oxygen and bCPAP in the community, anxiety and fear surrounding care before initiation of treatment, and a shift in beliefs to a more positive opinion of treatment regardless of whether their child lived or died (Table 2). Additionally, participants were able to identify potential benefits, harms, and alternatives to both oxygen and bCPAP.

Knowledge and feelings about oxygen and bCPAP prior to treatment

Despite longstanding use of oxygen in Salima District Hospital, knowledge of oxygen, and also bCPAP, was low within the participants' communities. Before participation in this study, only 13% (7/54) of participants personally knew someone who had received oxygen therapy. Despite limited personal exposure to oxygen, all mothers had heard of oxygen from members of their communities. Almost all information participants received from the community was negative. Eighty-three percent of mothers (45/54) explicitly, and without prompting, mentioned hearing negative opinions of supplemental oxygen therapy in the community, many using the phrasing that "oxygen kills" (Table 2).

> "At the village, people say that oxygen kills babies. So, when they told me that my baby will be on oxygen, I was very much worried about it." (oxygen, age 19, child survived)

They were also told to refuse therapy. One mother described her experience by saying,

> "People back in the village tell you to refuse the oxygen when you come to the hospital because they say a child who is put on an oxygen machine dies easily." (oxygen, age 33, child survived)

Not only are negative things said in the community, but also within the hospital. Mothers often reported being told to remove the equipment temporarily or permanently by other caregivers and this led to several self-reports of interrupting care.

> "When you are in that room, people stop what they were doing and start looking at you. Some [mothers] say 'I thought they are supposed to be removing it when the doctor is not there?'… They say because [bCPAP] kills." (bCPAP, age 33, child survived)

Some patients reported observing mothers abscond with their child. One mother also reported having her child taken off therapy by a relative.

> "One mother ran away with her sick child… She was afraid that oxygen kills. People were telling her that oxygen kills." (oxygen, age 24, child survived)

The knowledge about bCPAP was significantly lower; most reported they had never heard of bCPAP before being educated by the study staff at the hospital. However, those who had heard of bCPAP reported similar negative concern that it can worsen the child's condition or kill the child.

> "People in the community say that when a child is on bCPAP, they do not feed because the nose is covered and the child cannot swallow and they say it doesn't take long before the child dies" (bCPAP, age 35, child died)

In both groups, community beliefs led mothers to feel anxious and fearful prior to treatment initiation. Additional sources of fear included lack of familiarity with the machine and the severity of illness of the other children receiving treatment.

For peer review only

> "I was scared because it was my first time to see that thing and I was very worried... When I was entering the room, I found another child dying. So my feeling was that my child was going to die because that is the room where children die." (bCPAP, age 17, child survived)

When one mother was asked about the cause of her anxiety, she responded, "Primarily it was because of what people were saying. But since I had never seen this before, my worry multiplied." (oxygen, age 24, child survived)

Some women did not endorse anxiety around treatment. Instead, women in both groups referred to religious faith and trust in the medical providers as sources of comfort before treatment. These positive opinions were more common among the 13% of women who had had previous firsthand exposure to oxygen therapy.

> "I also did not mind what people were saying in the community… I said 'My child will receive this treatment; whatever happens to them; it is the will of God.'" (bCPAP, age 35, child died)

For peer review only

250 **Table 2:** Major themes that emerged

Community Knowledge	Overall low knowledge, with less knowledge about bCPAP compared to oxygen	"We are coming from the communities where we have no knowledge about this [bCPAP]."
	Negative thoughts about supplemental oxygen: "Oxygen kills"	"In our community, we are told that when someone is on oxygen they die so quickly because oxygen increases the death pace."
Feelings before Treatment	Almost all very anxious due to things said in the community or the appearance of the oxygen cannula or bCPAP machines and facemasks	"People say oxygen kills and this makes people feel afraid of the treatment. Someone ran away because she was afraid of the oxygen "
Alternative Treatments	Traditional medicine includes use of herbs and "traditional medicine tattoos"	"My granny tried to help my baby using herbs …She used some to rub herbs against their ribs, and some she made them drink, but it never worked out."
	Baby should be kept warm and environment clean	"The main cause of pneumonia is cold so if the child is kept warm it helps in that no coldness can penetrate them again."
	Injections or paracetamol can be given at health centers	"When a child has suffered from pneumonia and you go to the hospital, the doctors give the child injection."
Feelings after Treatment	Caregivers remained supportive of both oxygen and bCPAP regardless of child's outcome	"Oxygen helps but where the condition is not reversible, it does not. It takes someone to be lucky but it is helpful."
	Frequently stated that they would encourage others to seek treatment	"I am capable of encouraging them... I tell them that if a child has pneumonia, take them to the hospital and if they want to give them bCPAP, accept it."
Benefits to treatment	Helps the child breathe, allows child to feed and become more active, and aids in the child's survival	"I felt good because my child was able to feed while they had the oxygen tubes in the nose."
Harms to Treatment	Majority had no concerns, though some worried about bruising, rashes, and nasal injury and bCPAP machine malfunctions	"I don't think oxygen therapy can harm a child in any way because the oxygen that is found in the machine is the same that we breathe normally"
Education Timing	Majority prefer information to be given before treatment	"It is not practical to just put the child on bCPAP before giving information to the mother… people say that this thing kills and if that can happen, the mother can be so afraid and maybe she can run away."
	Desire for community sensitization	"I feel it is better to sensitize the people in the communities prior to their visits to the clinic because the people will have the information already."

251

For peer review only

Alternative treatments for the child's illness

Participants provided several examples of pneumonia treatments that have been used in the community. Many referred to traditional medicine techniques which consist of using herbs to burn and inhale the smoke, to rub on the baby's ribs, or to make drinks. Traditional healers may make "traditional medicine tattoos" in which herbs are placed beneath the child's skin via cuts or "tie the baby" which refers to making a necklace with a bag of grass and traditional medicine and placing it around the baby's neck or waist.

> "When we are at the village, we are told that the best way is to go to the traditional doctors. The traditional doctor makes traditional medicine tattoos and they also provide other medicines to drink so that the baby should be urinating frequently. After those medicines, they provide other medicines as well that they tie the baby. They say that when the baby is tied, it helps pneumonia to stop." (oxygen group, age 21, child survived)

While multiple alternative medicine techniques were endorsed, the majority of mothers who participated in the FGDs agreed that the best treatment is to seek care at a health center. Mothers who had tried traditional medicine before commented that they did not see improvement.

> "It is just very unfortunate that people go for traditional medicine but the best thing to do is to rush to the hospital." (bCPAP group, age 26, child survived)

Other proposed treatments for pneumonia included keeping the baby warm and medications such as antibiotics and acetaminophen.

Beliefs and feelings after treatment with oxygen or bCPAP

After treatment, the mothers were supportive of their care regardless of her child's health outcome and perceived multiple benefits of both treatments. Mothers whose child survived on oxygen or bCPAP expressed happiness and gratitude and they would recommend it to others.

> "My child was coming here when they were gasping...bCPAP recovered the child... It saved the life of my child and I will never forget. I will proclaim the benefits of bCPAP wherever I will go and I will encourage people to go for this treatment whenever they are offered." (bCPAP, age 35, child survived)

Mothers who had a child die while on therapy were still accepting of treatment. They often referred to other children who improved as evidence for its effectiveness.

> "Eventually my child died, not because they were on bCPAP but because it was time for them to die. So I cannot say that bCPAP kills just because my child did not survive; it is helpful because I saw other children surviving but I was just unfortunate that my child died." (bCPAP, age 30, child died)

Perceived benefits and harms of oxygen and bCPAP

The most commonly discussed benefit was that the machines help children breathe. Feeding was also often used as a marker of health with mothers referencing the ability to feed again as a benefit to both oxygen and bCPAP treatments. Once on treatment, their child became more active. Overall, mothers claimed that the machine helped their child to survive.

> "Oxygen is helpful. Even when a child is critically ill but when they put on oxygen the child is able to feed." (oxygen, age 38, child died)

> "I feel [bCPAP] increases the oxygen levels and help the child to breathe properly. If the child was not able to feed, they start feeding." (bCPAP, age 30, child died)

A majority of the mothers had no concerns about treatment. Some mothers were concerned about bruising, rashes, or nasal injury. Other concerns included machine malfunctions or electricity black outs, which are common in Malawi.

> "My only observation was that after receiving the treatment, my child had red skin meaning that the belt had caused some friction on the skin." (bCPAP, age 35, child survived)

Education about oxygen and bCPAP

Given the low community knowledge about oxygen and bCPAP, mothers were asked about the best time to be given information about treatment. The majority of mothers preferred information about the treatment to be given before any medical care was provided to the child, as was done in this trial, to address any concerns or allow the mother to decline treatment.

> "We are coming from the communities where we have no knowledge about this thing. What people talk about this thing is different from what is on the ground. So it is better to give the information about it before giving treatment so that people are well sensitized on how it works in order to deal with their fears they had from the community." (bCPAP, age 35, child died)

However, several felt it was more important to start the child on treatment first then provide information to the caregiver. This was often the case when they felt the child was very sick and treatment should not be delayed.

> "[With] how serious the child is; it is better to start treating the child before giving the information so that the child should get better." (bCPAP, age 26, child survived)

For peer review only

"If you have gone to the hospital sometimes the severity of the illnesses differs …I feel that the proper thing would be to start treating the child first with the oxygen therapy then they can start giving you the information." (oxygen group, age 24, child survived)

Another common theme was the desire for education in the communities. Mothers recognized a need for community sensitization to feel more comfortable about coming to the hospital.

"I would love if this information is given to us before the child gets sick so that when we come here, it should not be a strange thing to us. They can use the health surveillance assistants to sensitize the communities so that by the time the child will be sick, when we come here, we should not waste time getting the information but instead the child should start treatment right away." (bCPAP, age 26, child survived)

DISCUSSION

We found that many communities have negative beliefs and fears of supplement oxygen. In the communities, many women have been told that "oxygen kills" and offered alternative traditional medicine. They have been told to remove the oxygen therapy, which causes interruptions in treatment. Most caregivers were experiencing supplemental oxygen for the first time, and they reported feeling anxious mostly due to the community input. Regardless of the randomization group and outcome, after receiving treatment the caregivers reported satisfaction with the treatment and would even encourage others to go to the hospital for care. Mothers of children who died referenced temporary improvement in their child's clinical status or seeing other children improve with treatment as evidence for its effectiveness. This suggests that education and exposure to the treatments, even in the setting of a poor outcome, can be effective.

For peer review only

Many participants saw the benefits of oxygen and bCPAP care, and little saw harm aside from bruising or other marks from wearing the mask. Most mothers prefer information about treatment to be given before it is started and believe that the community would benefit from sensitization.

Oxygen therapy has been standard of care at SDH for children with severe pneumonia since Malawi implemented oxygen throughout the country beginning in 2000[3]. Despite availability for 18 years, mothers still reported widespread negative beliefs in their communities. However, mothers who had experienced oxygen firsthand reported entering the study with positive feelings. bCPAP had only been available at SDH through CPAP IMPACT for 2 years prior to these focus group sessions. Even though it was less familiar to mothers, those who had heard of it expressed similar fears and beliefs. Mothers of neonates receiving bCPAP in a NICU in Malawi expressed similar fears and anxieties.[11] Both studies demonstrated satisfaction with the treatment after initiation suggesting that exposure and education can reduce anxiety in mothers of children requiring respiratory support.

The perception that oxygen can kill children could lead mothers to delay seeking care and to seek other treatment options in their communities. Caregivers reported seeing traditional healers or trying home treatments before coming to the hospital. It is unclear how long caregivers delayed seeking more formal healthcare while under the care of traditional healers. They also reported feeling desperate when they arrived at the hospital and willing to accept any treatment given the condition of their child. If only the sickest children receive oxygen, mortality will likely remain high, which may continue to propagate negative beliefs among the community. Even once arriving at the hospital, we found mothers reported continuing to hear negative opinions from

other caregivers and were deterred from accepting or continuing treatment. The intermittent removal of the mask or nasal prongs by caregivers during oxygen and bCPAP treatments could interfere with treatment effectiveness and lead to poorer than expected outcomes. Caregivers implied that removal of oxygen and bCPAP treatments occurred when healthcare providers were not present, perhaps while caring for other children or at night when the staff to patient ratio was lower. While the frequency of removal was unclear, its occurrence is nevertheless alarming. These findings suggest that in the absence of more intensive and widespread community sensitization the use of oxygen and bCPAP in Malawi may be more effective in units with higher provider to parent ratios to facilitate closer monitoring of parental interactions with their children, rather than in general pediatric wards as was done in CPAP IMPACT. District hospitals in Malawi do not have high dependency or intensive care units.

Oxygen support has been shown to be critically important in the care of children with respiratory distress and is included in the WHO list of essential medicines[19,20]. For oxygen to have its full benefit and for implementation of newer technologies such as bCPAP to be effective, there is a clear need for community education. Caregivers displayed a strong support for both oxygen and bCPAP following treatment, even if their child died. This suggests that with exposure and education both can be considered acceptable therapies among caregivers. Caregivers in multiple groups expressed a desire for information in their communities before their children were sick. Community health workers (CHWs) have been shown to be effective in both community management of some diseases and preventative measures.[21] Community health workers delivering preventative care in LMICs have been found to be effective in community education.[22] Similar efforts may be effective in increasing education about respiratory support

technologies. Additionally, as many mothers commented how they would recommend these therapies to other mothers, mothers who have experienced treatment could be a useful resource for educating their communities.

Throughout focus group discussions, mothers referred to feeding as a surrogate marker for health. They described needing to bring the child to the hospital because they had stopped feeding. The ability of the child to eat was often cited as a benefit of therapy and a way of seeing improvement. There have been strong efforts in Malawi to encourage exclusive breast feeding and their child's ability to eat (breastfeed or solids for older children) was clearly important to mothers. This may potentially help explain some of the findings of CPAP IMPACT, which showed increased risk mortality in the bCPAP group, compared to the oxygen group.[15] The authors speculated that these findings could be in part due to a higher risk of aspiration from oral feeding despite recommendations for intravenous fluids or nasogastric feeds while receiving positive pressure ventilation from bCPAP, compared to low flow oxygen. The use of feeding as a marker of health status supports the concern that mothers may have continued to feed orally despite the child's decreased respiratory status and the provider's recommendation against oral feeding.

There are several notable limitations. First, the views of study participants may not be representative of all views. Mothers who participated in a group had already consented to having their child receive therapy and all focus groups were conducted post-intervention. Given we did not collect detailed demographic information from main trial participants, we cannot evaluate whether focus group participants were or were not representative of the main trial participants.

The near universal transition from a negative or fearful opinion to one of acceptance may suggest that only mothers who thought the therapy was beneficial chose to participate in focus group discussions, which would introduce bias into the results. These results should therefore be interpreted within this context. Additionally, groups did not include mothers who never presented to the hospital. This population is harder to reach and would require resources beyond the capacity of this study. However, participants were able to clearly talk about community views and opinions they had heard from their peers and the sample size of women included was sufficient to achieve thematic saturation. Second, the beliefs of participants may not be generalizable to other contexts and oxygen use and bCPAP implementation in other settings will need to consider unique culture viewpoints. Fewer women with deceased children participated in the focus groups. However, thematic saturation was achieved, and this group's views did not differ significantly from caregivers whose children survived. Lastly, in this setting, mothers may be compelled to provide answers that they believe are desired by the research team. We attempted to avoid this by explaining to mothers that all opinions were open and by choosing a facilitator who was independent of clinical care and had never met or interacted with mothers prior. Focus group meetings took place outside the hospital ward in a private room and only Malawians were present to encourage honesty and openness among participants.

CONCLUSION

In Malawi there are widespread negative beliefs about oxygen and bCPAP therapies with community members often citing that "oxygen kills babies." These beliefs may lead to delays in care for children with respiratory disease and may also interrupt care within the hospital. Mothers reported feeling anxious about allowing their child to receive treatment. Widespread

community sensitization is needed to educate communities and improve acceptance of oxygen and other respiratory therapies. Additionally, these results suggest that the role of feeding and its relationship to the effectiveness of respiratory therapies in Malawi needs further evaluation.

DECLARATIONS

Funding

KS received funding for this work through a Doris Duke Charitable Foundation grant (2016177) supporting the Doris Duke International Clinical Research Fellows Program at the University of North Carolina Chapel Hill. The Bill and Melinda Gates Foundation (OPP1123419), International AIDS Society (141022), and Health Empowering Humanity provided funding for CPAP IMPACT.

Conflicts of Interests

The authors declare that they have no conflicts of interests.

Author Statement

KS, LR, TM, and EDM participated in study design, data collection, and statistical analysis and were major contributors to manuscript writing. MT participated in FGD guide preparation, conducted the FGDs, completed translation and transcription, and contributed to analysis and manuscript preparation. DK contributed to data collection and design and implementation of CPAP IMPACT. MH, NL, AS, ME, and EDM contributed to study design and implementation of CPAP IMPACT. EDM was the principle investigator. All authors contributed to manuscript preparation and approved the final manuscript.

For peer review only

Acknowledgments

We thank caregivers and their children for participating in this trial, the Malawi Ministry of Health, and the CPAP-IMPACT study team for their contributions to this study.

Data Sharing

De-identified transcripts of FGDs are available on request from corresponding authors.

For peer review only

References

1. Troeger C, Forouzanfar M, Rao PC, et al. Estimates of the global, regional, and national morbidity, mortality, and aetiologies of lower respiratory tract infections in 195 countries: a systematic analysis for the Global Burden of Disease Study 2015. *The Lancet Infectious Diseases* 2017;17(11):1133-61.
2. Enarson PM, Gie RP, Mwansambo CC, et al. Reducing deaths from severe pneumonia in children in Malawi by improving delivery of pneumonia case management. *PloS one* 2014;9(7):e102955-e55. doi: 10.1371/journal.pone.0102955
3. Enarson P, La Vincente S, Gie R, et al. Implementation of an oxygen concentrator system in district hospital paediatric wards throughout Malawi. *Bulletin of the World Health Organization* 2008;86(5):344-48.
4. Nowadzky T, Pantoja A, Britton JR. Bubble continuous positive airway pressure, a potentially better practice, reduces the use of mechanical ventilation among very low birth weight infants with respiratory distress syndrome. *Pediatrics* 2009;123(6):1534-40. doi: 10.1542/peds.2008-1279 [published Online First: 2009/06/02]
5. DiBlasi RM. Nasal continuous positive airway pressure (CPAP) for the respiratory care of the newborn infant. *Respiratory care* 2009;54(9):1209-35.
6. Wilson PT, Morris MC, Biagas KV, et al. A randomized clinical trial evaluating nasal continuous positive airway pressure for acute respiratory distress in a developing country. *The Journal of pediatrics* 2013;162(5):988-92.
7. Walk J, Dinga P, Banda C, et al. Non-invasive ventilation with bubble CPAP is feasible and improves respiratory physiology in hospitalised Malawian children with acute respiratory failure. *Paediatrics and international child health* 2016;36(1):28-33. doi: 10.1179/2046905514y.0000000166 [published Online First: 2014/12/02]
8. Machen HE, Mwanza ZV, Brown JK, et al. Outcomes of patients with respiratory distress treated with bubble CPAP on a pediatric ward in Malawi. *Journal of tropical pediatrics* 2015;61(6):421-27.
9. Lum LC, Abdel-Latif ME, de Bruyne JA, et al. Noninvasive ventilation in a tertiary pediatric intensive care unit in a middle-income country. *Pediatric Critical Care Medicine* 2011;12(1):e7-e13.
10. Wilson PT, Benckert MM, Moresky RT, et al. Development and Implementation of a Training-of-Trainers Program for Continuous Positive Airway Pressure in Neonatal and Pediatric Patients in Five Low-and Middle-Income Countries. *Journal of tropical pediatrics* 2017;63(5):358-64.
11. Gondwe MJ, Gombachika B, Majamanda MD. Experiences of caregivers of infants who have been on bubble continuous positive airway pressure at Queen Elizabeth Central Hospital, Malawi: A descriptive qualitative study. *Malawi Medical Journal* 2017;29(1):5-10.
12. Peeler A, Fulbrook P, Kildea S. The experiences of parents and nurses of hospitalised infants requiring oxygen therapy for severe bronchiolitis: A phenomenological study. *Journal of Child Health Care* 2015;19(2):216-28.
13. Foster J, Bidewell J, Buckmaster A, et al. Parental stress and satisfaction in the non-tertiary special care nursery. *Journal of advanced nursing* 2008;61(5):522-30.
14. Cervantes AC, Feeley N, Lariviere J. The experience of mothers whose very low-birth-weight infant requires the delivery of supplemental oxygen in the neonatal intensive care unit. *Advances in Neonatal Care* 2011;11(1):54-61.

For peer review only

15. McCollum ED, Mvalo T, Eckerle M, Smith AG, Kondowe D, Makonokaya D, Vaidya D, Billioux V, Chalira A, Lufesi N, Mofolo I, Hosseinipour M. Bubble continuous positive airway pressure for children with high-risk conditions and severe pneumonia in Malawi: an open label randomized controlled trial. Lancet Resp Med. 2019; In press
16. Ritchie J, Spencer L. Qualitative data analysis for applied policy research. *The qualitative researcher's companion* 2002;573(2002):305-29.
17. Patton MQ. Qualitative research: Wiley Online Library 2005.
18. Strauss A, Corbin JM. Basics of qualitative research: Grounded theory procedures and techniques: Sage Publications, Inc 1990.
19. World Health Organization Model List of Essential Medicines, 21st List, 2019. Geneva: World Health Organization; 2019. Licence: CC BY-NC-SA 3.0 IGO.
20. Rojas-Reyes MX, Granados Rugeles C, Charry-Anzola LP. Oxygen therapy for lower respiratory tract infections in children between 3 months and 15 years of age. Cochrane Database of Systematic Reviews 2014, Issue 12. Art. No.: CD005975. DOI: 10.1002/14651858.CD005975.pub3.
21. Haines A, Sanders D, Lehmann U, et al. Achieving child survival goals: potential contribution of community health workers. *The Lancet* 2007;369(9579):2121-31.
22. Gilmore B, McAuliffe E. Effectiveness of community health workers delivering preventive interventions for maternal and child health in low-and middle-income countries: a systematic review. *BMC public health* 2013;13(1):847.

For peer review only

Appendix 1: Example facilitator guide for focus group discussions with mothers whose child was on oxygen

Thank you all for joining us for this discussion. You have been invited to participate in this discussion because your child participated in a study where they were randomized to receive either bubble CPAP or oxygen therapy for severe pneumonia. We wanted to better understand your thoughts on treatment for pneumonia in young children and your experiences with oxygen.

1. Had you seen or heard about oxygen therapy before your child was hospitalized at Salima District Hospital?
    a. If so, please discuss what you had seen or heard.
    b. Had you heard about bubble CPAP, the other treatment used in the study?
2. What is your understanding of what oxygen therapy is?
    a. Where did you learn about oxygen therapy?
        i. PROBE: Can you identify who at the hospital provided you with information or was able to answer your questions?
    b. Do you feel like you received enough information or teaching about oxygen therapy?
        i. When did this teaching occur?
        ii. PROBE: Did you learn about oxygen therapy before or after your child was started on treatment?
3. How did you feel about your child receiving oxygen therapy at the beginning?
    a. What concerns did you have?
        i. PROBE: Did you hope your child would be in the oxygen therapy or bubble CPAP arm of the study? Why?

For peer review only

   b. Did your feelings about oxygen therapy change during the hospitalization?

      i. PROBE: Can you describe what it felt like to see your child on oxygen therapy?

4. What are the benefits of oxygen therapy for your child?

   a. PROBE: Can someone give me an example of a way that oxygen therapy may benefit a child with pneumonia?

5. Do you have any concerns that oxygen therapy can harm your child?

   a. If so, please explain.

   b. Do you think others in the community would have concerns about oxygen therapy?

6. What are other types of treatments for pneumonia?

   a. Are they better or worse? Why?

Thank you for your participation in this discussion today. This information will help us design future educational materials to help providers talk to mothers and caregivers about bubble CPAP and treatment for severe pneumonia. Please let us know if you have any questions. *[Clarify any misconceptions if any came up during the discussion.]*

For peer review only

Reporting checklist for qualitative study.

Based on the SRQR guidelines.

Instructions to authors

Complete this checklist by entering the page numbers from your manuscript where readers will find each of the items listed below.

Your article may not currently address all the items on the checklist. Please modify your text to include the missing information. If you are certain that an item does not apply, please write "n/a" and provide a short explanation.

Upload your completed checklist as an extra file when you submit to a journal.

In your methods section, say that you used the SRQRreporting guidelines, and cite them as:

O'Brien BC, Harris IB, Beckman TJ, Reed DA, Cook DA. Standards for reporting qualitative research: a synthesis of recommendations. Acad Med. 2014;89(9):1245-1251.

		Reporting Item	Page Number
**Title**			
	#1	Concise description of the nature and topic of the study identifying the study as qualitative or indicating the approach (e.g. ethnography, grounded theory) or data collection methods (e.g. interview, focus group) is recommended	1
**Abstract**			
	#2	Summary of the key elements of the study using the abstract format of the intended publication; typically includes background, purpose, methods, results and conclusions	3
**Introduction**			
Problem formulation	#3	Description and signifcance of the problem / phenomenon studied: review of relevant theory and empirical work; problem statement	5

For peer review only

Purpose or research question	#4	Purpose of the study and specific objectives or questions	5
**Methods**			
Qualitative approach and research paradigm	#5	Qualitative approach (e.g. ethnography, grounded theory, case study, phenomenolgy, narrative research) and guiding theory if appropriate; identifying the research paradigm (e.g. postpositivist, constructivist / interpretivist) is also recommended; rationale. The rationale should briefly discuss the justification for choosing that theory, approach, method or technique rather than other options available; the assumptions and limitations implicit in those choices and how those choices influence study conclusions and transferability. As appropriate the rationale for several items might be discussed together.	6
Researcher characteristics and reflexivity	#6	Researchers' characteristics that may influence the research, including personal attributes, qualifications / experience, relationship with participants, assumptions and / or presuppositions; potential or actual interaction between researchers' characteristics and the research questions, approach, methods, results and / or transferability	6
Context	#7	Setting / site and salient contextual factors; rationale	6
Sampling strategy	#8	How and why research participants, documents, or events were selected; criteria for deciding when no further sampling was necessary (e.g. sampling saturation); rationale	6
Ethical issues pertaining to human subjects	#9	Documentation of approval by an appropriate ethics review board and participant consent, or explanation for lack thereof; other confidentiality and data security issues	7
Data collection methods	#10	Types of data collected; details of data collection procedures including (as appropriate) start and stop dates of data collection and analysis, iterative	7

For peer review only

		process, triangulation of sources / methods, and modification of procedures in response to evolving study findings; rationale	
Data collection instruments and technologies	#11	Description of instruments (e.g. interview guides, questionnaires) and devices (e.g. audio recorders) used for data collection; if / how the instruments(s) changed over the course of the study	7
Units of study	#12	Number and relevant characteristics of participants, documents, or events included in the study; level of participation (could be reported in results)	8
Data processing	#13	Methods for processing data prior to and during analysis, including transcription, data entry, data management and security, verification of data integrity, data coding, and anonymisation / deidentification of excerpts	7
Data analysis	#14	Process by which inferences, themes, etc. were identified and developed, including the researchers involved in data analysis; usually references a specific paradigm or approach; rationale	7
Techniques to enhance trustworthiness	#15	Techniques to enhance trustworthiness and credibility of data analysis (e.g. member checking, audit trail, triangulation); rationale	7
**Results/findings**			
Syntheses and interpretation	#16	Main findings (e.g. interpretations, inferences, and themes); might include development of a theory or model, or integration with prior research or theory	8
Links to empirical data	#17	Evidence (e.g. quotes, field notes, text excerpts, photographs) to substantiate analytic findings	8
**Discussion**			
Intergration with prior work, implications, transferability and contribution(s) to the field	#18	Short summary of main findings; explanation of how findings and conclusions connect to, support, elaborate on, or challenge conclusions of earlier scholarship; discussion of scope of application /	16

For peer review only

		generalizability; identification of unique contributions(s) to scholarship in a discipline or field	
Limitations	#19	Trustworthiness and limitations of findings	19
**Other**			
Conflicts of interest	#20	Potential sources of influence of perceived influence on study conduct and conclusions; how these were managed	21
Funding	#21	Sources of funding and other support; role of funders in data collection, interpretation and reporting	20

The SRQR checklist is distributed with permission of Wolters Kluwer © 2014 by the Association of American Medical Colleges. This checklist was completed on 24. September 2019 using https://www.goodreports.org/, a tool made by the EQUATOR Network in collaboration with Penelope.ai

For peer review only
